# Convention of Professors of the Medical Colleges

**Published:** 1867-06

**Authors:** 


*Convention of Professors of the Medical Colleges.*—From the following account of the proceedings of the Medical Convention, held last month in Cincinnati, it will be seen that the Medical Colleges are following the lead of the Dental Colleges in the effort to elevate the standard of Collegiate Education.

In pursuance of a call of a committee appointed by the American Medical Association, at its last session, held in Baltimore, May 3, 1866, delegates from most of the medical institutions of the country met in Cincinnati, in the faculty room of the Medical College of Ohio.

Professors Holloway, ot Louisville; Davis, of Chicago; Donaldson, of Baltimore; Blackman, of Cincinnati; and March, of Albany, were appointed a committee to report on the order of the different subjects which were to occupy the attention of the Convention.

After which the Convention adjourned to 4 o'clock P. M.

In the afternoon session this committee reported the following distinct propositions for the consideration of the Convention.

"1. That every student applying for matriculation in a Medical College shall be required to show, either by satisfactory certificates or by a direct examination by a committee of the faculty, that he possesses a thorough knowledge of the common English branches of education, including the first series of mathematics, elements of natural science, and sufficient knowledge of Latin and Greek to understand the technical terms of the profession, and that the certificates presented or that the results of the examinations thus required, be regularly filed as a part of the records of each Medical College.

"2. That every medical student be required to study not only three full years, but also to attend three regular annual courses of Medical College instruction before being admitted to an examination for the degree of Doctor of Medicine.

"3. That the *minimum* duration of an annual lecture term, or course of medical college instruction, shall be five calendar months.

"4. That every medical College shall embrace in its curriculum at least thirteen professorships, including substantially the following branches, namely: Descriptive Anatomy, Physiology and Histology, Inorganic Chemistry, Materia Medica, Organic Chemistry, Toxicology, General Pathology and Public Hygiene, Surgical Anatomy and Operations of Surgery, Medical Jurisprudence, Medical Ethics, Practice of Medicine, Practice of Surgery, Obstetrics and Diseases of Women, Clinical Medicine and Clinical Surgery. That these several branches shall be divided into three groups or series, corresponding with the three years required for medical study: The first or freshmen series, shall embrace Descriptive Anatomy, Physiology and Histology, Inorganic Chemistry and Materia Medica. To these the attention of the student shall be mainly restricted during the first year of his studies, and on them he shall be thoroughly examined by the proper members of the faculty at the close of his first course of Medical College instruction, and receive a certificate indicating the degree of his progress. The second or junior series, shall embrace: Organic Chemistry and Toxicology, General Pathology, Public Hygiene, Surgical Anatomy and Operations of Surgery, Medical Jurisprudence, and Medical Ethics. To these the attention of the medical student shall be directed during the second year of his studies, and on them he shall be examined at the close of his second course of Medical College instruction the same as after the first. The third, or senior series, shall embrace Practical Medicine, Practical Surgery, Obstetrics and Diseases of Women, with Clinical Medicine and Clinical Surgery in hospital. These shall occupy the attention of the student during the third year of his medical studies, and at the close of the third course of Medical College attendance he shall undergo a general examination in all the departments, as a prerequisite for the degree of Doctor of Medicine.

"The instruction in the three series of branches is to be given simultaneously, and to continue throughout the whole of each annual college term; each student attending the lectures on such branches as belong to his period of progress in study, in the same manner as the Sophomore, Senior and Junior classes each pursue their respective studies simultaneously throughout the collegiate year, in all our literary colleges.

"5. That the practice of selling individual tickets by members of medical college faculties should be abolished, and, in the place of it, each student should be charged a specified sum for each annual course of medical college instruction; the sum being the same for each of the three courses before graduating: and any student or practitioner who has attended three full courses in any one college, shall be entitled to attend any subsequent course or courses in that college gratuitously. The fees paid for each annual course of college instruction should be paid to the Treasurer of the college, and subsequently distributed to each member of the Faculty at such time and in such proportion as the Trustees and Faculty of each college shall determine.

"6. That every Medical College should immediately adopt some effectual method of ascertaining the actual attendance of students upon its lectures, and other exercises, and at the close of each session of the attendance of the student a certificate, specifying the time and the courses of instruction actually attended should be given, and such certificate only should be received by other colleges as evidence of such attendance."

The resolutions were adopted. Professor Davis then introduced the following resolution:

"*Resolved*, That a committee of five be appointed by the President, whose duty it shall be to present the several propositions adopted by the convention to the trustees and faculties of all the medical colleges in this country and solicit their definite action thereon, with a view to the early and simultaneous practical adoption of the same throughout the whole country; and that the same committee be authorized to call another convention whenever deemed advisable."

The Chair appointed the following gentlemen that committee: Prof. Davis of Chicago, Donaldson of Baltimore, Gross of Philadelphia, March of Albany, Blackman of Cincinnati. Thereupon on motion of Professor Hammar, the convention adjourned subject to the call of the committee.

